# The Present State of Microscopical Science, Medically Considered

**Published:** 1859-05

**Authors:** John H. Packard

**Affiliations:** Philadelphia


ART. II.—*The Present State of Microscopical Science, Medically Considered.* By JOHN H. PACKARD, M.D., of Philadelphia.

ALTHOUGH nearly two centuries have elapsed since the publication of the first really scientific observations made with magnifying glasses, the full value of this method of research has been developed only within the last twenty-five years. Both as an optical instrument, and in its mechanical accessories, the compound microscope seems now to be hardly susceptible of further improvement. Of all the means at our command for extending our knowledge of the physical world, there is, perhaps, none that opens a wider or more attractive field of inquiry, since it discovers to the eye a new realm of life, animal and vegetable. Interesting as these minute organisms are in themselves, they are doubly so in a physiological point of view; for in them the laws of vitality are set forth under the simplest conditions. Moreover, the intimate structure of the human frame, in health and disease, can now be thoroughly investigated; so that in regard to anatomy, physiology and pathology, which together constitute the true foundation of philosophical medicine and surgery, the student at the present day enjoys advantages not granted to his predecessors.

The enthusiasm of the votaries of a new science is apt to lead them into claiming for it greater importance than is sustained by subsequent experience; and when its novelty wears off, and it is found not to supersede other and older methods of investigation, it is in danger of unmerited mistrust and neglect. There may be some source of fallacy in the subject of study; inexperienced, inaccurate or prejudiced observers may commit palpable errors; or too much may be expected of the means employed; all which circumstances must be taken into account in estimating the worth of any branch of inquiry.

The object of the present paper is to discuss the value of the microscope as a means of medical research; to examine into the reliability of its testimony, and the extent of its range. For this purpose, it is necessary to review what has been accomplished by its aid, and to survey the still debatable ground.

Let it be distinctly understood in the first place, that the microscope cannot set aside other sources of information. Its powers are confined to the recognition of certain qualities of matter, unappreciable by the naked eye; in other words, it simply aids the sight. Hence, just as the grosser appearances either of healthy or diseased tissues are without meaning, unless subjected to careful analysis and comparison, just as the closest reasoning must be employed in their study, so also with the characters revealed by the microscope. In matters of sight alone, the eye, whether assisted or not, must be depended upon; but when there arise questions of interpretation, when the value of differential signs is to be decided, or the meaning of a fact to be explained, other evidence becomes necessary. Microscopy, therefore, like other sciences, comprises two essential elements; one being the mere collection of facts or observations, and the other the grouping of these and the deduction of laws from them. All men are, to a greater or less extent, observers; very few are philosophers. The mere addition of another optical instrument to the one supplied by nature, cannot do away with the necessity of careful and discriminating thought; it simply increases the facilities for gathering material. Should the medical profession henceforth devote themselves solely to microscopy, it would be as if all other branches of industry were abandoned for the mere digging of gold.

To form an idea of the influence actually exerted by the revelations of the microscope upon the various departments of medicine, it is only requisite to glance over the publications of the first quarter of this century, and then to take up those of the present day; the difference cannot but be manifest.

How, then, has this change been brought about? Nothing has contributed more to its production, to the increase in accuracy of anatomical description, and to the clearer understanding of the processes observable in health and disease, than the discovery of the *cell*, as the primary organic form. And since this discovery is the basis of the fundamental law of structure, with which, as microscopists, we are particularly concerned, it will be well to take a brief survey of the conditions and general bearings of this law, before entering upon the subject of its special applications.

The limit between the organic and the inorganic kingdoms is now clearly drawn. Crystallization, the faint shadow of the living *form*, stands near the line, but below it. *Vitality, past or present, and a definite compound structure,* belong to all true organisms. The microscope cannot reveal the nature of that Promethean fire, which the Creator of all dispenses at his will; but it lays before us the material envelope, the outward and visible sign of that mysterious endowment. It shows us the developmental changes which result in the perfect tissues, and the successive steps by which those tissues are again prepared for resolution into their original elements, as well as the alterations in them which constitute disease.

With the physiological question of a vital force, as distinct from all other known agencies, we are not here concerned; it may be that the exciting cause of organization is a special and undefinable influence, or a mere combination of certain electrical, chemical and mechanical conditions; but we find that an organizable material is indispensable, and we find also, that wherever organic forms appear, they do so secondarily; in other words, that there is no such thing as spontaneous generation. Carbon, hydrogen, oxygen and nitrogen, with heat, moisture and electricity, are not the sole requisites for the development of an organic form; the assimilative power of a previously existing *germ* is absolutely essential.

Under the influence of such a germ, the organizable material may acquire either one of several forms, differing only in appearance. It may become a cell, a mere nucleus, a fibre, or a tube, or it may be utilized without taking on any of these characters, as in the "matrix" of cartilage, or the "basement-membrane" of epithelial layers. What the mutual relations of these forms may be, as rudimental, transitional, completed or retrograde stages of the same portion of matter, will also come under consideration in speaking of the development of the various tissues in which they occur.

Connected, however, with the very idea of the typical cell, are the questions of the function and date of its nucleus. The typical cell, be it understood, is composed of a wall, inclosing the cell-contents, of which the nucleus and its nucleolus form a part; and the point now at issue is, whether the nucleus appears first, and the cell is developed around it, or, the cell being completed, a portion of its contents is arranged so as to form the nucleus. Several facts go to favor the idea embodied in the first-named view, that the nucleus is the true germ of each cell; thus, in all rapidly-growing tissues, free nuclei are apt to abound, and the nuclei contained within cell-walls are apt to be very large; in the cells of tissues which need constant renewal, as in muscle, the nuclei do not disappear, as they often do in those which have attained a permanent character; and in cells which multiply by division, such as those of cartilage, each segment carries with it a portion of the nucleus.

One other theory of cell-formation might be contended for, viz., that the origin of the cell and that of its contained nucleus are simultaneous; the cell then undergoing a further process of development, while the nucleus remains stationary. But if the cell and its nucleus are called into being at the same time, the former is often imperceptible, while the latter seems perfected; so that, for practical purposes, the nucleus is still the prime agent in the growth of the cell.

As to the nucleolus, its significance is not appreciable; it is not by any means so constant as the nucleus, although its presence and vesicular appearance have been proposed as diagnostic signs of malignancy, by some pathologists.

Schwann's description of the formation of the cell around the nucleus, is better than any since given. (*Mikroskopische Untersuchungen*, Berlin, 1839.) "On the outer surface of the nucleus there is deposited a layer of matter separated from the surrounding blastema. This layer is not at first well defined, externally, but becomes so by the addition of fresh molecules. The layer is of variable thickness, and may be either homogeneous or granular. No distinction exists, as yet, between the wall of the cell and its cavity. The deposition of new particles of matter between the former, still continues; so that the whole layer, if it is thin, or its outer portion, if it is thick, at length constitutes a membrane. The commencement of this consolidation of the outer part of the layer into a membrane, may be observable soon after its external limit becomes well-defined; but, usually, the membrane is not to be clearly made out until later, while it becomes thicker and more sharply defined within; in many cells no distinct membrane exists, but they seem solid; only their outer part seems to be more compact than the rest.

"The cell-wall, once consolidated, grows by the continual deposit of new molecules between those making it up, and also by a process of intussusception; it thus becomes separated from the nucleus. The interval between the two is, at the same time, filled with fluid, which is the cell-contents."

Now, in this doctrine of a cell, with its formation and subsequent developments, we have the ultimate analysis of anatomy. Each cell, in any living organism, presents the phenomena of life; birth, growth, assimilation, reproduction and death, occurring to each, in due order and time. A certain aggregation of various forms of cells constitutes an animal or a plant; the aggregation of their functions constitutes its physiology, and the partial derangement of their functions, its pathology.

Here, then, we undoubtedly have a key to the laws of structure; from the humblest unicellular plant to the complicated organism of man, the *cell* is the form under which life manifests itself. Life without structure is an inconceivable idea to the human mind, with its present capacities, and therefore it never enters into our practical calculations. But if structure and life are inseparable, if, moreover, there can be traced certain relations between the characters of a cell and its function or life-action, then the cell-doctrine has a wide and most important application. This idea will be developed further as we advance in our subject; but let it be here remarked, that to the microscope is due the recognition of the cell, and that by the microscope alone can the cell be studied.

The matter of a classification of ultimate structural elements is at present undecided, mainly because it is still a vexed question whether or not pathological and normal forms are essentially identical. That such a classification will eventually be made, we firmly believe; until it is made, and all known structures are grouped under definite heads, microscopy cannot claim rank with other branches of natural science. Nor is the value of minute anatomy compromised by this statement, since its study has been so recently commenced, and its range is so wide, that its resources are as yet undeveloped.

As medical men, our main subject of inquiry concerns the difference between health and disease, in order that we may preserve the former condition or arrest the latter. And here the idea may be suggested, that, perhaps, abnormal phenomena are more intimately connected with structural changes than is generally supposed. For instance, in diarrhœa, dependent upon a so-called "weakness" of the mucous membrane of the intestine, the epithelial lining of the tube may not improbably be altered in its character, either as a cause or as an effect of the morbid state. The "irritations" to which so many forms of suffering are ascribed, may be due to or productive of changes in texture, appreciable to the aided sight. In fact, the line of division between functional and organic disease is not, by any means, distinctly drawn; possibly, no such line exists.

By thus extending the term "organic," so as to include in it all departures from the normal type of the state or function of cells, we define more clearly the limit between health and disease, in theory at least. If the connection between the material state of a tissue and its function is not inseparable, then there may be such a phenomenon as purely functional disease; if otherwise, this term is meaningless.

In other words, we would suggest that the working of the entire machinery composing the living organism is dependent for its accuracy upon that of the individual tissues by which it is made up. And however temporary any derangement may be, however superficially it may seem to lie, it appears a reasonable supposition that it is the result of abnormity in material structure. Take the case of functional disease of the heart; can any one assert that there is no such thing as an abnormal condition of the cells composing the brain or spinal cord, or of the blood, manifested just in this way? Again, in functional disease of the brain or spinal cord; is there any absurdity in the idea that it denotes some organic alteration, temporary or permanent, as the case may be, in the structural state of some other part?

Such a theory cannot include all mental disorders; we do not carry our materialism into the domain of psychology. So far as bodily phenomena are concerned, directly or indirectly, as causes or as effects, we would explain them upon the principle now stated. And if this theory be correct, then the cell-pathology proposed by Virchow, as the true combination of solidism and humoralism, finds a rational basis.

One other general question may be mentioned here, without anticipating its future discussion in connection with special points. An opinion is held by many pathologists that all pathological forms are either retrogressions, transformations, or repetitions of typical, physiological structures. The decision of this question, as has been already stated, is essential to the classification of ultimate structural elements, since it involves the position to be assigned to very important growths. If there does not exist any type of cell-formation different from the physiological, then the so-called malignant deposits, for instance, must be conformable to the latter. But if so, they must either depend for their malignancy upon some condition extrinsic to their microscopic elements—a supposition fatal to cell-pathology—or a power must be conceived to be inherent in certain cells, of acquiring a new function and signification, without losing their identity. In the former case, the local manifestation is a mere accident, remarkably constant it must be confessed,—the cell does nothing; in the latter, the cell is possessed with a sort of evil spirit, and is transformed from a quiet and useful member of the body corporate, into a most inveterate disturber.

That the local deposit is not the sum of the morbid condition, is evident from the general symptoms accompanying it; that the constitutional vitiation, the diathesis, is not all, is shown by the constancy of its topical manifestation. The precise connection between the two we cannot define; but it seems philosophical to suppose that they have the same origin, and the same essential nature. It must, however, be conceded that the constitutional affection is *sui generis;* and why it should be denied that the local deposit is likewise peculiar, does not appear.

Having now taken a brief survey of the great truth revealed by the microscope,—the existence and universality of the organic cell,—and having discussed in general terms the bearing of this recognized fact upon physiology and pathology, let us proceed to the more special examination of our subject.

We have to deal with (1) *minute anatomy;* (2) *progressive changes;* (3) *processes.* Each of these, again, has reference (1) to the state of *health;* (2) to that of *disease.*

Our researches must, therefore, have a sixfold character; and they can only be made practically useful by bringing the several divisions now laid down into mutual connection; in other words, by looking at them successively in regard to each tissue. The following arrangement of our subject will, therefore, be adopted :—I. The special application of the cell-doctrine, or the discussion of the microscopic elements of healthy tissues generally. II. The individual tissues, including their normal anatomy, their physiological functions as connected with this, and their pathological changes. III. Such processes as are open to microscopical study, and yet involve several textures. IV. Such structural elements as constitute morbid deposits. V. Matters secreted and excreted. VI. Animal and vegetable parasites.

I. *The microscopic elements of the healthy tissues generally*, have been alluded to in connection with their typical form ; and we have now merely to discuss their mutual relations, and the practical importance of these.

It must be borne in mind that matter, whether organized or not, is constantly acted on by physical forces ; and that although these may not fully explain the phenomena met with, they must be taken into account. At all events, they have a modifying influence ; and it is more philosophical to make the best use of what we know certainly, than to appeal to the vague and indefinite. Thus, it may be stated that the primordial organic form, the cell, is guided in the alterations it undergoes by the mechanical pressure it sustains ; that its spherical shape is the result of an equilibrium between the cohesive and repulsive powers of its particles ; and that when it becomes elongated, polygonal, or constricted, the change is due to those particles moving in the direction of least resistance. An increase of the cell-contents by endosmosis of liquid from without must of course render the pressure from within outward more powerful in every direction, and therefore can only distend the sphere equally ; their diminution by exosmosis will, on the contrary, favor the action of external forces. In regard to some points, this theory is sufficient ; thus the flattened shape of the superficial layer of epidermic cells is fully accounted for in this way. But to explain the development of some other tissues, as for instance striated muscular fibre, some less purely mechanical theory is requisite. Hence, although this law is never superseded, it will not answer as the general principle of progressive change. The precise nature of the force originating and guiding this phenomenon is beyond the province of sight, and the microscope can therefore reveal its effects only.

Assuming then the fact of some immaterial agency, we find that the tissues are by its influence developed from an ovule, which "*possesses the nature and constitution of a simple cell.*"* By its segmentation this becomes a mass of cells, and these, by their own changes and by their assimilative power over the organizable material, build up the textures of

* Kölliker's Microscopical Anatomy, Am. ed., p. 642.

the animal frame. In the whole course of this process the nucleus (primarily the *germinal vesicle*) appears to exert an indispensable influence; it may be called the medium through which the immaterial agent manifests itself.

But the very performance of any function by a cell involves an expenditure of its powers of life; hence the constant presence of effete matters in the organism; and as the organizable matter consisted of certain chemical elements in combination, so, having completed its term of life, it is resolvable again into the same chemical elements. This resolution may occur within the body, or the cells may be first cast off by a process of excretion; in the former case the chemical elements may be discharged under very different conditions of combination from those which they presented at the outset.

We find, therefore, within the organism, as soon as the formative process is so far advanced that functions are assignable to its several portions, matter in very various states. Nutritive material, tissues at different stages toward completion, and the products of life-action, must be present. The first of these is simply the liquor sanguinis, or plasma; the third includes secreted and excreted matters. And among the matters excreted we find some, such as carbonic acid and water, which are ordinary chemical compounds; others, such as the coloring matters of the bile, which are derivable only from the animal economy; and lastly, worn-out structural elements.

But it is with the second state of matter that we have now to deal—with the nuclei, cells, fibres, tubes, and the so-called *structureless membranes*, which go to make up the body.

Free *nuclei* exist physiologically only in the chyle at an early stage, and here they soon surround themselves with a wall, forming the chyle-corpuscle or cell. The idea that the red blood-corpuscle is a free nucleus is no longer tenable since the publication of Mr. Wharton Jones's researches. In morbid growths, nuclei sometimes elongate into fibres; but this probably never occurs as a healthy process.

*Cells*, as such, are found floating in the blood, chyle, and lymph; they lie definitely arranged upon mucous and serous surfaces, and in cartilage and bone; they constitute the contractile tissues, and the nerve-centres; and the accidental injuries to which the animal body is subject are repaired by means of them. In this latter process, an analogy with the original formation of the living being is clearly traceable.

*Fibres* are met with in connective tissue, in tendons, ligaments, the periosteum and perichondrium, in the dura mater, and in the sheaths of the nerves, in the sclerotic coat of the eye, the cornea, the capsule of the lens, the skin, and the matrix of certain cartilages. They are never primary forms, but are derived either from cells or from nucleated blastema; in the latter case it is not improbable that there is really a cell-structure present, but less evident than usual.

*Tubes* occur as elementary forms in the sheaths of nerve-fibrils; they are also found, as the result of the coalescence of cells, in the capillary networks of the lymphatic and blood-vessel systems.

*Structureless* or *basement-membranes* are expansions of organized material, which, as their name implies, present no indication of either of the foregoing types of structure; their importance has been, perhaps, too little insisted upon. Their extent is very great; they underlie all the mucous and serous epithelia, separating the proper cells from the connective tissue which sustains and binds together the vessels so as to constitute the submucous and subserous areolar textures.

Now it seems as if there were an evident analogy, if not identity, subsisting between the cell-wall, the fibre, the tube, and the *basement*-membrane. They are all, strictly speaking, equally destitute of structure, as far as we can ascertain; the difference in aspect observed in them being altogether due to the different *mechanical* requirements which are to be fulfilled. As regards the cell-wall, the tube, and the membrane, their functions are plainly limitary wherever we find them; while the office of fibres is no less clear. Nor does it need any further proof than has been already given, that the origin of all these is the same.

If these views be correct, the matter actually composing the living body is susceptible, theoretically, of a threefold division, into (1) nuclei; (2) limitary or mechanical substance; (3) cell-contents. Perhaps it may be added, that the peculiarities manifested by different sets of cells depend entirely upon the character of their contents, of their nuclei, or of both; but this point may be more suitably discussed in connection with special histology.

As to the age attainable by the structural elements now reviewed, this, of course, varies greatly. It is probable that there is a continual substitution of new molecules for those expended in the performance of function, throughout the entire economy. Some parts, as for instance the epidermis, are exposed to influences which wear them away; others, like bone, are apparently permanent; in either case, provision is made for a more or less abundant supply of fresh material, in the form of nutriment. How long a time is required for the entire destruction and renewal of the human body by this molecular substitution, cannot be accurately estimated, although an idea prevails that such a process is completed every seven years. The period must vary both in the individual tissues and in the aggregate of them, according to several conditions; such as the time of life, differences of constitution or of external circumstances, and the accidental influences of disease.

Another question occurs here, as to the mode of multiplication or new formation of cells in the living body. The primordial cell, the ovum, as has already been said, divides and subdivides into many segments, each of which becomes a complete cell; but this method is in the adult animal almost confined to the growth of cartilage. Most commonly the new cells arise in a blastema derived from the blood, under an influence exerted by the adjacent tissue; without which *assimilative* force it would seem impossible for their development to be begun. Gemmation or budding is not seen in any of the cells of the higher organized beings, nor is it probable that the formation of cells within cells is carried on beyond the period of embryonic life, in the healthy tissues.

So far, we have dealt with normal histology only; it may, however, be stated here, that we find in morbid deposits the same general elements as in the healthy tissues; in other words, they are amenable to the fundamental laws of structure. The discussion of this subject belongs, of course, to a later portion of this article.

II. To take up thoroughly the study of *the several tissues, including their normal anatomy, their physiological functions as connected with this, and their pathological changes,* would be to fill volumes. Our aim must therefore be to give a brief, but discriminating *resumé* of the established or disputed doctrines in regard to the main topics comprised under the above heads; and even this will occupy so much space, that many minor trains of thought must be left to the reflection of the reader.

In order to discuss the tissues systematically, some definite arrangement of them is desirable. And as we are especially concerned, at present, with the visible characters observable in them, it would seem most proper to found our classification upon the peculiarities of structure revealed by the microscope. But, as a certain relation exists between form and function, and as, moreover, we claim for microscopy an auxiliary office only, we may legitimately draw upon other sources of information, just as other methods of study are greatly indebted to ocular investigations for a similar purpose. Without so doing, the only classification that could be offered would either be vague and inadequate, or consist in a mere enumeration of organs. I would therefore propose the following arrangement, hoping to justify its propriety and convenience in the course of the ensuing discussion:—

1. The MECHANICAL tissues, including
   (*a*)—The *passive:*
      Limitary membrane,
      Bone,

Cartilage,

Fibrous tissue, } Fibro-cartilage,

Adipose tissue;

(*b*)—The *active:*

Striated muscle,

Non-striated muscle.

2. The NERVOUS, including
Corpuscles of nerve-centres,
Commissural filaments.
3. The GLANDULAR, including
Serous membranes,
Mucous membranes,
Ductless glands.
4. The OPTICAL, including the
Cornea,
Lens,
Hyaline membrane,
Membrane of Jacob.
5. FREE CORPUSCLES, including those of the
Blood,
Chyle,
Lymph.

I have based the above classification upon the most convenient peculiarity of each tissue; it is by no means intended to designate the sole, or even the most important functions pertaining to its items. All the elements of the body are, however, embraced in it, under separate heads, and in each of these, except the last of all, a function is stated or implied. We shall take them up in the same order.

(1) The MECHANICAL tissues, as their name implies, are concerned in the production or transmission of mechanical force, or in that resistance by which alone such force can be utilized.

(*a*) Among the *passively* mechanical tissues, the first place belongs properly to *limitary membrane.* The importance and extent of this so-called *structureless* expansion of organized matter may be estimated from the fact that, as before stated, it constitutes the cell-wall, and the boundary of all surface-layers of cells; and that it is probably identical with most, if not all the true fibres. The term *tissue* is perhaps incorrectly applied to it, since its atoms are so arranged as not to present any visible interweaving. Since, however, it plays a very important part in the animal economy, it may be allowed to rank with its co-workers; and since its only apparent office is a mechanical one, it is fitly placed in this connection. I would suggest the equal impropriety of the term *structureless* as applied to this form of matter, which in fact is fundamental to all structure; the word *basement*-membrane is far more expressive of the true position to which it is entitled. One fact would seem to prove its purely mechanical function: wherever any other duty is performed, there is added some substance by way of cell-contents, to which the action may be fairly ascribed.

It is through this limitary membrane, whether in the form of a cell-wall or a basement-membrane, that the liquids pass in the phenomenon of endosmosis and exosmosis. And according to the views now prevailing,* this action must be looked upon as altogether mechanical; at least the membrane itself exerts no elective influence. So long, indeed, as the law of indivisibility of matter remains in force, the mere passage of the liquids must take place through pores, and hence the solid particles of the basement-membrane must be arranged in a definite order. It is therefore evident that there exists, so to speak, a system of microscopic hydrostatics and hydro-dynamics, and that a very important function of the membrane in question is carried on under the provisions of this system. Supposing, indeed, the regulation of the interchange of liquids to be the sole office of basement-membrane, what could be more absolutely essential to the proper working of the animal economy?

From what has now been said, it may be seen that the discussion of this subject must spread itself throughout the whole of the present article. I would simply add here, for the sake of completeness, that the cell-walls of morbid deposits do not seem to differ in any respect from those of the normal structure; and that the only change which basement-membrane undergoes in disease, is a breaking down, either from defective supply or quality of nutritive matter, or from a loss of assimilative power.

The microscopic history of *bone* is of great value, since it affords a very striking illustration of the degree to which cell-transformation may take place. The originally nuclear character of the lacunæ, and the hardening of the cell-contents by deposition of earthy matter, are evident from the examination of sections made from bones at different stages of growth. And the fact that bone may be formed out of either fibrous tissue, cartilage, or cells, (to be further dwelt upon in connection with some morbid deposits,) has great significance as bearing upon the relation existing between cells of apparently diverse character. In the study of the repair of fractures, the microscope has been of essential service, showing the analogy which exists between this process and the healing of injuries of the soft parts; the primary state of the reparative material is found to

* These views are admirably set forth in Dr. Draper's "Human Physiology," chapter vii, on "Absorption by the Blood-vessels."

be either that of nucleated cells or of nucleated blastema, the secondary, that of cartilage or fibrous tissue.

Mollities ossium is shown by the microscope to be a degenerative change in the bony texture, or, in other words, a defect of nutrition; and, as will be further insisted upon, this fatty deterioration is amenable to the very same laws governing it in the other tissues.

Ranking structurally with the bones, and allied to them in their morbid states also, are the teeth. Here we find the cement, or tooth-bone, closely resembling the common osseous tissue; the dentine presenting a marked analogy, in its wavy canaliculi, to those seen diverging from the lacunæ in a thin section of the fibula; the enamel, the most earthy of all the compounds of the economy, still bearing the same typical impress in its form, origin, mode of nutrition, and diseases. It is difficult to conceive why the sum of these structures, the tooth, should be separated from its analogue in form and office, the bone. No difference whatever exists between the two, except in the mode of origin of the tooth from the papilla of the dental groove; but this is entirely similar to that of a flat cranial bone. And although in so authoritative a work as Todd and Bowman's "Physiological Anatomy," the teeth are said to be developed "from the mucous membrane* covering the dental arches, and not from the maxillary bones," whence the inference is drawn that they "seem to correspond with the tegumentary appendages of animals, such as horns, nails, feathers, and not to be a portion of the true osseous system;" the reason assigned does not appear to justify the setting aside of such important analogies. Here the relationship, doubtful to the naked eye, is fully established by microscopic investigations.

*Cartilage* is a structure accessory, as it were, to bone; wherever found, it is either a transitional stage of ossifying matter, or supplementary to the harder parts of the skeleton. Nerves are not found in it at any time, nor are vessels, except during its formation or ossifying stages. Cartilage presents two processes of cell-multiplication—that by division of complete cells, and that by endogenous formation of young cells. The *matrix* of cartilage seems analogous in many respects to basement-membrane; it sometimes shows no visible trace of structure, but often has a dimly granular, or even fibrillated appearance.

Now this matrix may have a more important significance than would at first appear. This granular or fibrillated character may be assumed to be the shadow of an actual fibrous texture; and thus is established a rela-

---

* This is surely a very loose expression; no *mucous membrane* could ever give origin to bone, or tooth-structure, although such a formation might readily occur in the fibrous tissue underlying it.

tionship at least between the substance of the matrix and the substance of the fibre. But the matrix undergoes a transformation into the wall of the bone-cell; so that here we have matrix, fibre, and cell-wall virtually identical. The subsequent change of the latter by the deposition of earthy matters is, so to speak, a mere accidental circumstance, and therefore does not impair the force of the fact in reference to the laws we are now endeavoring to illustrate.

The only pathological changes of a simple character met with in cartilage, are its thickening and its atrophy. It is often the case that minute oil-globules are visible within the cells of apparently healthy cartilage, and it may be that fatty degeneration of cartilage is a common constituent of the ulceration and other diseases to which the joints are liable. But this subject greatly requires further elucidation, and calls for very careful and discriminating study, owing to the many sources of error in both the observation and appreciation of phenomena.

Some hints have been already thrown out in regard to the histological position of *fibrous* tissue. No doubt can exist as to the very close alliance between the *white* and *yellow* fibres; the difference between them probably lies in their chemical elements, and is non-essential, at least for our present purpose. According to the researches of Dr. Leidy, as published in his edition of Sharpey and Quain's Anatomy, the origin of both kinds of fibres from nucleated cells can be clearly made out in the embryo; and we can sometimes, by the addition of acetic acid, render visible the nuclei of the white variety when fully formed.

In the transformation of the original cell into the fibre, of whichever kind, the nucleus and the cell-contents seem to become equally useless. There is no evidence to show that the nucleus, when brought into view by means of acetic acid, retains any functional activity; while the fact that in many instances no nucleus can be detected, would favor the idea of its presence being merely accidental. Nor can the fibre be so treated as to give any sign of a central cavity, either with or without contents.

Here then we have, it is evident, an extremely low organic form—a mere extension of material, bearing the same relation to the limitary membrane before described, as flax twine to a linen fabric; and assigned to this form an extremely low function—the lowest, perhaps, of any. Neither in white fibrous tissue nor in yellow is any force generated, of any kind, unless it be capillary attraction upon their nutritive fluid. Low, however, as the function of fibres may be, it is nevertheless of vast importance in the economy; there is not a single organ which would without their aid be available, either for protection, support, or connection.

Occupying this humble position, fibrous tissue is actually exempt from all disease, except inflammation, and even this does not induce any pathological change in its microscopical characters. I may be allowed here to remark upon the wisdom of the provision, by which so wide-spread and indispensable a constituent of the frame is of such a nature as to be undisturbed by the derangements of the higher textures.

A change sometimes observed, but only becoming pathological by disturbing some other tissue in its function, is the conversion of fibrous tissue into bone. Thus the coracoid ligament may become completely ossified, still retaining the grosser outlines of its primary condition; showing that there is nothing peculiar in the fibrous tissue from which the teeth and cranial bones are developed, but that cells, fibres, bone, and cartilage are very intimately allied, if not absolutely identical.

Such an inference might also be drawn from the existence of *fibro-cartilage*, in which the matrix is plainly of a fibrous character. In this we have only a more marked degree of the fibrillation alluded to as often observable in the simple cartilages, and particularly distinct, according to Dr. Leidy, in those of the articulations.

*Adipose* tissue, besides its mechanical use in the prevention of injurious pressure, plays a very important part in the maintenance of temperature. Neither of these functions is recognizable by the microscope, which, however, exhibits the extremely simple character of the tissue in question; the oily matter being confined within cell-walls, as contents, and, according to Todd and Bowman, a nucleus being present in every such cell in the early stage of its existence. This is perhaps the very lowest form of cell; it is certainly the least essential, and multiplies or disappears the most readily. Fat may be abnormally deposited, but is not itself liable to morbid change.

Next in order come—

(*b*) The *actively* mechanical tissues, which might have been called the *contractile* tissues, on account of their inherent and distinguishing property of shortening under the influence of stimulation. But the anatomical peculiarities presented by them beneath the microscope are equally distinct from those of all other tissues, and moreover serve to divide accurately the two varieties from one another. The close alliance between striated and non-striated muscle is evident from the identity of their primary condition in the embryo. Wherever a quick and energetic action is requisite, be it voluntary or involuntary, there we find the striated form; wherever, as in most of the involuntary muscles, the contraction is to be gradual and sluggish, the non-striated. The precise nature of this contractility is absolutely unexplained, in either; but the idea would seem plausible that the property resides in the *cell-contents* in each case. The division between the several cells constituting a fibre of the striated variety is imperceptible, although the application of acetic acid, by bringing out their nuclei, affords us a trace of their position. And I would suggest that perhaps the undulatory outline of a contracting fibre, sometimes so very regular, marks that of the individual cells in their linear arrangement; in which case the contraction may be described as an increase of the equatorial diameter of each cell at the expense of its polar.

Now this contractility may be easily seen to be altogether independent of nervous influence, by watching an isolated fibre under a magnifying power of 250 diameters. Such a fibre, taken from the leg of a living frog, may remain quiescent, but sometimes it may be seen to contract in the manner commonly described. Again, it will often begin to darken, its transverse striæ will *disappear*, and its outline will become irregular, while its direction may be variously altered; numerous *longitudinal* lines, short and without definite arrangement, previously visible, become more distinct. As far as I have yet observed, this change always commences at the free ends of the fragment, and not at any intermediate point.

Microscopic investigation has of late years established the existence of non-striated muscular fibres in many parts formerly considered to depend for their contractility upon some other provision, or perhaps to have no expulsive power upon their contents. Among these parts may be mentioned the skin, the prostate, the urethra, and the trabecular tissue of the spleen; the muscularity of which the microscope has placed beyond a doubt.

We are also indebted to the microscope for the settlement of another question, connected with the subject of reproduction. The muscular fibres of the uterus, having fulfilled their office in expelling the fœtus, undergo a physiological fatty degeneration; just as militia-men at the close of a war are very apt to become worthless members of society. Such a change occurs in unstriated muscle elsewhere, as a pathological process; as for instance in the coats of arteries, and in the intestinal walls. It may take place also in the striated variety, affecting the heart more frequently than any other portion of it.

Allied to this species of degeneration, and often existing in connection with it, is a peculiar deposit which has been termed *fibrinous*. This presents the appearance of fibro-plastic or elongated cells, together with some granular and fatty matter; it has been observed chiefly in the heart's walls, occupying sometimes the whole and sometimes only a part of their thickness. The neighboring muscular structure is often found to have undergone a conversion into fibrous tissue, either coincidently with the deposit or as a later stage of it; while intermixed with the deposit are muscular fibres, some in a state of fatty degeneration, some merely broken up, and some entirely unchanged. The boundary line between the healthy and diseased tissue is in some places well marked, in others indistinguishable. Often the two layers of pericardium exhibit adhesions such as exist in the pleura just over a spot of hepatization in a lung affected with pneumonia, and fibrinous clots are apt to occupy the corresponding endocardial surface. Similar fibrinous deposits are commonly present in the spleen, probably in connection with the smooth muscular fibre of its trabeculæ, in cases of this kind.

Here then we have an analogy drawn, by means of microscopic observation, between the two forms of muscle; they are proved to be allied in their origin, in their most essential property, and in their degenerative changes; and this fact affords a key to some physiological phenomena, as well as to some diseased states. Moreover, we have an additional instance of cell-development and metamorphosis; so that the idea of a sameness, not only in origin but in structural type, between the various tissues, is here again carried out.

(2) NERVOUS tissue presents a peculiar interest, in that it constitutes the medium of communication between the material and the immaterial. To enter upon the discussion of its physiology, would be to overstep the bounds of microscopy, nor could it be fairly dealt with in the space here allowed us.

We must, however, claim for the miscroscope the merit of having assigned to the nervous system accurate boundaries; of having shown the cellular character of certain portions, and the tubular or commissural character of others, and thus of having rendered essential aid in the investigation of the material agents by which the inscrutable processes of sensation, volition, and motion are carried on.

Some points have yet to be cleared up in reference to the minute anatomy of this portion of the frame. As to the mode of origin of the tubules at the nerve-centres, although some may very probably, as asserted by Kölliker, Robin and others, be prolongations of the filaments of caudate nerve-cells, yet according to Valentin tubules may be seen forming loops within the vesicular matter; and this latter view is supported by a case of monstrosity observed by Lonsdale.* Now if, as seems most likely, both these views are correct, I would suggest that they may combine to explain certain phenomena of nervous action; that all voluntary movements may be due to an influence exerted through the cells of the cerebro-spinal centre upon the nerves arising from them, while reflex action is carried on by the looped fibrils; each loop being the terminal part of an afferent, and the commencing part of an efferent fibril.†

---

* Quoted in Todd and Bowman's Physiol. Anatomy, from the Edinburgh Med. and Surg Journal, No. 157.

† I hope to be able to investigate this point more fully at some future time.

Again, the mode in which the ultimate nerve-fibrils are disposed beneath sentient surfaces is a matter still *sub judice*. It is asserted by some high authorities that they end in loops; by others, that their extremities are free; and by others again, that both these arrangements are observable. Connected with this point is another, in regard to the nature and office of the Pacinian corpuscles. The idea which perhaps finds most favor is, that these bodies are formed of concentric layers of a substance resembling the cell-wall, the innermost one inclosing the terminal portion of the nerve-tubule; but why or wherefore such a provision exists, is as yet unknown.

Microscopic examination has shown the existence of a layer of gray nerve-cells, forming the outer lamina of the retina at its posterior part. Hence the optic nerves are merely commissural; and hence also we have an explanation of the fact that gastric irritation may give rise to muscæ volitantes, diplopia, etc., this *portion of the sensorium* partaking in the disturbance. May not this circumstance account also for the frequency of ocular spectra in the various forms of delirium?

(3) No department of physiology is more indebted to microscopical research, than that of SECRETION. (This term is here used in its widest sense, and in our classification the word *glandular* was employed as co-extensive with it.) The material requisites for the process are, a vascular supply, a limitary membrane, and one or more laminæ of epithelial cells. How it is that one mucous or serous tissue differs from another,—why one should separate the constituents of urine and another those of saliva, cannot with our present methods be determined. Except in the case of the liver, and in that of the so-called blood-glands, the structural elements above enumerated are demonstrable in all secreting organs; and even in those just named, an *analogous* arrangement may, I think, be considered to exist.

These general statements are now to be illustrated and defended by individual instances.

*Serous membranes*, as every medical tyro knows, are shut sacs. They do not shed their epithelial cells perceptibly, but nearly always have only a single layer of them, which is very probably persistent; merely undergoing such molecular change as is inseparable from the performance of any function. The peritoneum, pericardium, pleuræ, arachnoid, endangium, tunica vaginalis testis, synovial membranes, and bursæ, although varying greatly in their extent and importance, and in the degree of wear and tear to which they must submit, are structurally identical. And the propriety of placing them under the head of *glandular* tissues will further appear when certain pathological conditions are brought under review.

In *mucous membranes* there is always either a free surface, or an outlet by which the cast-off cells are removed. The substratum of connective tissue is looser here than in serous membranes, and the epithelial cells form layers upon the surface of the basement-membrane. Now if we begin with the mucous lining of any gland, say of the parotid, we may trace a perfect continuity, through the skin, of every part of the *mucous epithelial system* with every other part. An exception to this statement would seem to exist in regard to some of the intestinal glands; but they do not in reality present any deviation from it.

Hence one epithelial system, including the skin and all the mucous membranes, constitutes as it were the surface-layer throughout the body; while another is made up of a number of shut sacs, the serous membranes, which exist independently of the former and of one another. The epithelial cells which line serous cavities are invariably of the squamous variety; those found upon mucous surfaces are squamous, columnar, or intermediate, and may or may not be furnished with cilia. In some glands, as for instance in the lactiferous tubes of the breast, and in the tubuli uriniferi, the cells are more evidently spherical than elsewhere. The serous membranes might have been ranked with the mechanical tissues, but for their structural characters; since the office they fulfill is simply the prevention of undue friction. But not only is this end attained by means of a process within the general definition of secretion; several facts go to prove the alliance subsisting between the two forms of epithelial tissue. Thus portions of uriniferous tubules, becoming closed, dilate into sacs having all the appearance of membranes originally serous; and at the fimbriated extremity of the Fallopian tube, a mucous membrane is continuous with a serous.

Now if, moreover, we consider the relations subsisting between the lymphatic and blood-vessel systems, we must see that the term *endangium* should include the lining membrane common to both, thus signifying a serous expansion of very great extent. And if the relation of this membrane to the mucous lining of the intestine be properly appreciated, the endangium would seem to derive blood-material, the mucous membrane secretion-material, by a process of interchange. Perhaps it may even be asserted, that the blood is in a manner secreted from the food and drink taken into the alimentary canal.

Of the matters separated from the blood, some assume forms the presence of which seems indispensable to the accomplishment of their allotted purpose,—as in the seminal liquid; others, like the biliary constituents, continue amorphous. The minute anatomy of the secreting membranes does not afford any clue to the cause of these variations; the question is only carried a step further back when we ascribe them to some difference in the contents of the cells.

Secretion is, therefore, reduced to this : certain substances being placed upon either side of a septum, made up of two layers of basement-membrane, with an epithelium on each free surface, an interchange goes on between them ; the nature of the interchange depending upon that of the cell-contents, or perhaps upon that of the nuclei, of the epithelium upon one side. Did our space permit, the further evolution of this idea, with a review of its special applications, would bring up some very interesting topics for discussion ; but it must for the present be postponed. Here, however, we must claim for the microscope the credit of having shown the precise character of the anatomical elements employed in secretion, and of having thus paved the way to greater accuracy of thought and expression than could otherwise have been attained ; and we must hope that more extended observation, particularly in microscopical chemistry, may afford a still clearer insight into the mysteries of this very important process.

The pathological changes undergone by serous membranes are confined to inflammation and morbid deposits, and will be discussed hereafter. Mucous membranes are sometimes affected with a local excess in the production of cells, constituting in part the growths known as epitheliomata. They are also, the skin especially, liable to diseases of which we know very little except the external appearance. In some of these, animal or vegetable parasites are discoverable by means of the microscope ; but whether as cause or effect, or as a mere coincidence, has not yet been determined.

Perhaps no class of affections has more completely baffled the attempts of etiologists than those just mentioned ; a fact to which may be ascribed the vague nomenclature and the empirical treatment very often adopted in regard to them. A series of well-directed microscopical observations upon this subject, although not promising a complete solution of the problem, might possibly either supply new data, or shed light upon facts already known.

Upon the subject of the *ductless glands* we have not much to say, the questions concerning them belonging much more to physiology at large than to microscopy, although sometimes originating with the latter.

The *spleen*, the largest and most readily investigated of all these bodies, has received most attention from microscopists. The principal point of discussion in regard to it is its relation to the blood-corpuscles,—whether it is the nursery or the cemetery of those bodies. Among German physiologists, Kölliker adopts the latter view, Gerlach and Virchow the former. These authors base their opinions upon microscopic observations made by themselves ; Kölliker's view is sustained by Mr. Gray, in his work on the spleen, on like grounds. Most American physiologists favor the idea that the spleen is a blood-forming organ, but are influenced rather by certain general reasons than by the visible phenomena.

But this question, as well as the anatomy, physiology and pathology of the *thymus and thyroid bodies* and *supra-renal capsules*, would require too much space, and would bring in too much irrelevant matter, to be discussed here. All these points are of great importance, doubtless; but what is known concerning them is at present in so crude a state as to forbid any attempt at generalization. Nor can we hope that here the microscope alone will clear up matters, although we may assert that, aiding and aided by close and careful reasoning, it will show itself not only useful, but indispensable.

(4) The term *optical* has seemed to me best adapted to designate certain elements found in the eye; some of them have an office partly mechanical, but their transparency, with or without refracting power, is their main peculiarity.

Microscopic research has demonstrated the nature of these parts to a great degree; the existence of a layer of epithelium on the posterior surface of the cornea, on the inner surface of the capsule of the lens, and on that of the membrane inclosing the vitreous humor, would seem to justify the idea that three serous membranes exist within the globe of the eye.

The cornea and hyaline membrane are shown to consist of fibres, whose peculiar transparency prevents their interfering with vision, while the mechanical support they afford is of essential importance. With regard to the crystalline lens, some difference of opinion still exists; Kölliker, Gerlach, and other German micrographers, consider its structure as tubular, while Todd and Bowman describe it as fibrous. Robin adopts the former view.

Great attention has been paid to the study of the membrane lining the concave surface of the retina, first described by Jacob in the Philosophical Transactions for 1819, and named after him. This *bacillar* layer may perhaps be looked upon as a modification of the columnar epithelium elsewhere met with, or it may be ranked with the fibrous tissues; its great value consists in its refracting power, and I would suggest that amaurosis may sometimes be due to the loss of this property. Again, supposing an analogy to exist between this structure and the lens,—an idea borne out by the views of some anatomists,—an alliance would thus be shown between some forms of amaurosis and cataract. But to establish the correctness of such a supposition would demand careful research, in the face of serious practical difficulties.

Upon other points in the pathology of the eye, deeply as this subject has been studied for centuries, we may yet hope to derive much that is valuable from well-directed investigation with the aid of the microscope. Very little has hitherto been attempted in this field.

(5) Of all the matters subjected to the scrutiny of science, few possess a higher interest than those "vital fluids" which we have now to glance at in their microscopical aspect. Constituting in fact one mass of circulating material, the chyle, lymph and blood are intimately allied; by some authors it is asserted that no difference exists between them except in degree of development.

According to this latter view, the chyle-corpuscles pass into lymph-corpuscles, and the majority of these again into red blood-disks, which, having run their course, are disposed of in some manner not yet positively ascertained. Its opponents, among whom is Dr. J. C. Dalton, of New York, consider the red corpuscles as persistent forms, essentially differing from the white, which are derived from the lymph. Dr. Dalton states also, in his recent work on Physiology, that the white globules are destitute of nuclei, and denies the existence of a cell-wall in the red disks; in these points we think very few observers will agree with him. And since no theory equally satisfactory is proposed as a substitute for the one thus rejected, the view expressed in connection with the subject of secretion, as to the origin of the blood-elements, seems to us the correct one.

Many difficulties are in the way of a direct solution of the questions which arise in regard to these matters. To trace the chyle, with its molecular base, along the mesenteric lymphatics, to watch the appearance in it of the white corpuscles, to follow these along through the thoracic ducts into the venous system, into the lungs, and then into the general circulation, to observe the gradual metamorphosis into the red blood-disk, is possible only in imagination. But when the aided eye has carefully marked the appearances presented by the circulating fluid at different points, reason combines these data with others, and the chain may be completed.

Comparative anatomy is here of great service. It seems to be in favor of the idea that the red disk is a cell which has no longer a perceptible nucleus; since in many vertebrata the coloring matter is contained in the interspace between the nucleus and the cell-wall. The study of the blood in the very early stages of life has also a high degree of interest. The blood originates, like all the other structures, from the plastic matter constituting the contents of the primitive cell or ovum; and just as muscular fibre contracts, or synovial membranes secrete, so the blood-disk at once assumes its function of carrying oxygen.

The office of the white corpuscle, unless it be to pass into the red disk, has not yet been pointed out. The microscopic appearances of the circulation, and the relations of the two forms of corpuscles, will be considered when we speak of processes.

But one pathological condition of the blood has been clearly made out by microscopical examination. It is claimed as a discovery both by Bennett and Virchow, and consists in an excess of the white corpuscles over their normal amount. We must, however, look behind this for the essence of the disease; something is wrong in the process of formation of the blood. Nor can we help regarding this circumstance as a confirmation of the idea that the white corpuscle is a developmental form of the red disk; but the precise starting-point of the deviation from health has yet to be assigned.

The blood-corpuscle-holding cells, described first I believe by Virchow as occurring in clots of extravasated blood, and especially frequent in the human brain after apoplexy, do not seem to have any important pathological signification.

Statements have been from time to time put forth, that alterations in the microscopic characters of the blood result from the action of various poisons; and Dr. Joseph Jones, in a paper published in Vol. viii of the Smithsonian Contributions, gives cuts of the precise change of form observed in the blood-cells of animals dying in an atmosphere of hydrogen. Many of these statements are extremely vague and unreliable; and the source of the error in other cases may be found in the action of water or other menstrua upon the cell-wall or its contents.

Having now reviewed rapidly, but we hope not superficially or unprofitably, the present state of microscopic anatomy, we must postpone the consideration of the remaining portions of our subject until another occasion. Our aim is not only to state what *has been* done, but to suggest what *is to be* done, in the hope that the position of microscopical science may be improved as well as vindicated and strengthened.

